# Editorial: Examining Mechanisms via Which Traumatic Stress Leads to Post-traumatic Stress Disorder Using Animal Models: Advantages, Pitfalls, and Future Directions

**DOI:** 10.3389/fnbeh.2022.966147

**Published:** 2022-07-04

**Authors:** Dayan Kessler Knox, Esther L. Sabban, Shigeru Morinobu

**Affiliations:** ^1^Department of Psychological and Brain Sciences, University of Delaware, Newark, NJ, United States; ^2^New York Medical College, Valhalla, NY, United States; ^3^Department of Psychiatry and Neuroscience, Hiroshima University, Hiroshima, Japan

**Keywords:** post-traumatic stress disorder, fear, extinction, transcriptome, epigenetics, neuropeptide Y, anxiety, arousal

Animal models of psychiatric disorders are powerful research tools that allow for the identification of circuits, molecular processes, and behavioral mechanisms through which psychiatric disorders manifest. These models are particularly powerful when they are used to identify how neurobiological phenomena contribute to specific symptoms within a psychiatric disorder (Insel and Landis, 2013).

Post-traumatic stress disorder (PTSD) is a debilitating mental health disorder which has a tremendous economic burden associated with it (Kessler et al., 1995, 2005; Kessler, 2000; APA, 2016). A better understanding of PTSD represents a core research interest for clinicians and basic scientists worldwide. In this regard, animal models can provide a, “basic framework,” for how aberrant circuit, molecular, and behavioral processes contribute to specific PTSD symptoms.

In this Research Topic, “Examining mechanisms via which traumatic stress leads to post traumatic stress disorder using animal models: advantages, pitfalls, and future directions,” a group of established stress researchers describes experiments that examine critical and relevant topics to PTSD. These include lateralized stress effects on medial prefrontal cortical (mPFC) modulation of emotion regulation (Canto-de-Souza et al.), stress effects on phase coupling between the mPFC and BLA (Wang et al.), transcriptome and epigenetic phenomena that are sensitive to traumatic stress (Ding et al.; Nwokafor et al.), social boding as a buffer against traumatic stress effects (Lee et al.), and sex differences in reactivity to traumatic stress (Knox et al.; Nahvi et al.; Zoladz et al.).

Using animal models to examine core PTSD does not come without challenges. Perhaps one of the greatest challenges for the field of traumatic stress research concerns differences in susceptibility to traumatic stress between female and male organisms. Virtually all animal models of PTSD fall short of advancing the field in this regard, as the susceptibility to traumatic stress that is observed in female humans is not typically recapitulated (if at all) in PTSD animal models. Three of the articles in this Research Topic address this (Knox et al.; Nahvi et al.; Zoladz et al.), but questions remain. Do the models need to be adjusted in order to observe effects? Do different stress models need to be utilized in males vs. female organisms? Do different behavioral tests need to be used in male vs. female organisms in order to better capture how traumatic stress leads to effects in males vs. females? A major push of future traumatic stress research should be to better explain sex differences in traumatic stress sensitivity as it pertains to PTSD.

## Author Contributions

All authors listed have made a substantial, direct, and intellectual contribution to the work and approved it for publication.

## Conflict of Interest

The authors declare that the research was conducted in the absence of any commercial or financial relationships that could be construed as a potential conflict of interest.

## Publisher's Note

All claims expressed in this article are solely those of the authors and do not necessarily represent those of their affiliated organizations, or those of the publisher, the editors and the reviewers. Any product that may be evaluated in this article, or claim that may be made by its manufacturer, is not guaranteed or endorsed by the publisher.
